# Macaque monkeys learn by observation in the ghost display condition in the object-in-place task with differential reward to the observer

**DOI:** 10.1038/s41598-018-36803-4

**Published:** 2019-01-23

**Authors:** Lorenzo Ferrucci, Simon Nougaret, Aldo Genovesio

**Affiliations:** 1 Department of Physiology and Pharmacology, Sapienza University of Rome, Rome, Italy; 2 PhD program in Behavioral Neuroscience, Sapienza University of Rome, Rome, Italy

## Abstract

Observational learning has been investigated in monkeys mainly using conspecifics or humans as models to observe. Some studies attempted to clarify the social agent’s role and to test whether non-human primates could learn from observation of a non-social agent, usually mentioned as a ‘ghost display’ condition, but they reported conflicting results. To address this question, we trained three rhesus monkeys in an object-in-place task consisting of the presentation of five subsequent problems composed of two objects, one rewarded and one unrewarded, for six times, or runs. Three types of learning conditions were tested. In the individual learning condition, the monkeys performed the first run, learned from it and improved their performance in the following runs. In the social and non-social learning conditions, they observed respectively a human model and a computer performing the first run and learned by the observation of their successes or errors. In all three conditions, the monkeys themselves received the reward after correct choices only. One-trial learning occurred in all three conditions. The monkeys performed over chance in the second run in all conditions, providing evidence of non-social observational learning with differential reward in macaque monkeys using a “ghost display” condition in a cognitive task.

## Introduction

Social learning is a major field of interest in neuroscience and it is in continuous evolution since the pioneering work of Bandura^1^. The research efforts have aimed to understand the neural bases of the differences between individual and observation learning. The ability to draw useful information not only through direct personal experience but also through the observation of conspecifics is a skill shared among several species, from human^2,3^ and non-human primates^4–10^ to invertebrates^11,12^ through birds^13–15^, rats^16^ and reptiles^17^. Studies on non-human primates demonstrated their ability to cooperate and monitor actions of a conspecific^18,19^. Moreover, previous works highlighted the ability of macaque monkeys to learn from human choices^20^, to monitor their goals^21–25^ and even to learn token values from their observation^26^.

However, despite an increasing number of behavioral studies in monkeys focusing on social learning, little attention has been given to its non-social form. Is the conspecific or the human partner necessary to enhance learning? Is it possible to learn by observation in a non-social context, such as observing a computer performing a task? The “ghost display” condition^14^ was defined in an experimental setting in which the experimenter moved a manipulandum that gave access to food without being seen by the animal. In such a condition, the manipulandum appeared to move by itself, without the intervention of an external physical agent. Comparing the role of social and non-social agents within the same task allows a better understanding of the role of the type of agent in the observational learning process. A number of studies have addressed this question using great apes or children^27–29^ and reported contrasting results. The ability to learn in a “ghost display” condition depended on the complexity of the movement being executed, on the experimental parameters, and on the nature of the task, e.g. the utilization of a tool, a sequence of movements or a simpler association between choice and action. The only study that examined such behavior in macaque monkeys provided a negative result^5^. The authors reported that rhesus macaques can benefit from the observation of a conspecific in a cognitive choice task. Nevertheless, monkeys were unable to extract useful information in the same paradigm but without the social partner, just observing and receiving feedbacks. Learning was present only after the observation of the expert monkey which performed the task sitting in an adjacent chamber. Taken together, all these findings suggest that observational learning in monkeys occurs only under appropriate conditions. While monkeys’ ability to learn by observation, from a conspecific or a human model, seems to be an established fact, it is still unclear whether non-social forms of observational learning can occur. We designed an experimental paradigm to compare three different forms of learning: individual learning, when the monkey exploited a trial and error strategy to understand which of two stimuli is associated with a reward; social observational learning, in which the monkey observed a human model perform the task; non-social observational learning, where instead of a human model the monkey observed the choices of “computer model”, in a ghost display condition. In all three versions of the task, the reward was delivered to the monkeys after a correct choice only. Consequently, when we refer to “observational learning” in the present manuscript, we refer to a special case in which the monkeys observed the choice performed by the human model or the computer and received themselves the reward.

## Results

### General aspects

**Figure 1:**
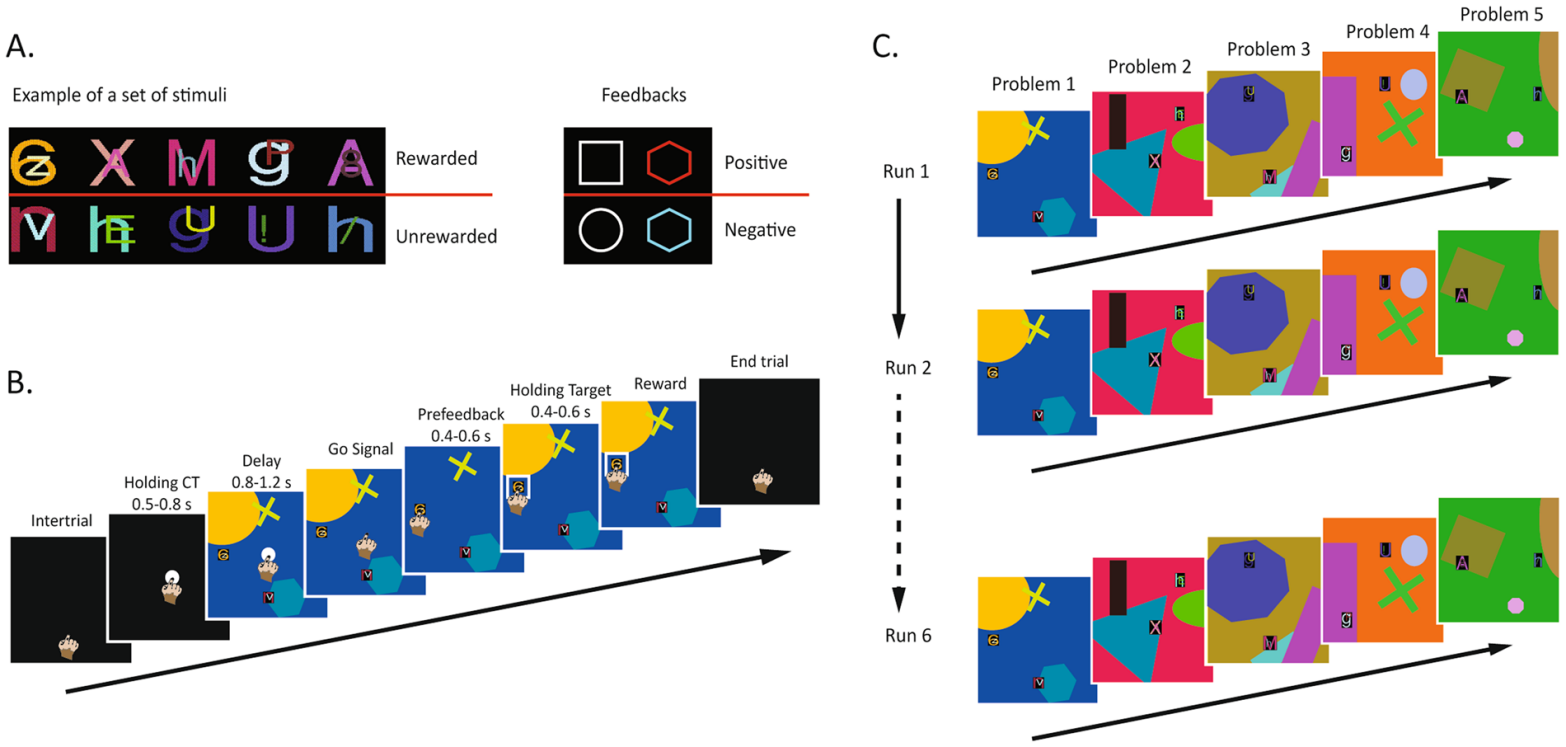
Task. (**A**) Examples of stimuli displayed as objects in a problem and stimuli displayed as feedback around the chosen object. (**B**) Example of the temporal sequence of a trial. The periods of the trial were identical in the three different conditions MA, HI, and CI. In the CI condition, the central target was red instead of white only in the first run. (**C**) Example of temporal sequence of the six runs. Five problems composed the first run and were subsequently represented in the same order for six times. Six runs composed one complete session.

Three monkeys performed three different versions of the object-in-place task (OIP; see Materials and Methods for detailed description). In this task, a trial began with the presentation of a central target (CT) on a touchscreen that the monkey had to touch to make a scene appear. A scene was composed of different geometrical figures and two different objects, one rewarded and the other not, composing the problem the monkeys had to solve. The monkeys had to perform five different problems to complete the first run of the task. After the first run, the second run started, with the same five problems presented in the same order, and so on until six runs were completed (Fig. 1). The three versions of the task differed with regard to the actor who performed the first run. In the monkey alone (MA) version, the first run was performed by the monkey, in the human interaction (HI) version by a human agent, and in the computer interaction (CI) version by the computer. In any case, the monkeys performed the remaining five. The data were collected on a total of 60 sessions for each monkey in each of the three versions of the task (MA, HI, CI) for a total amount of 540 sessions and 16 200 trials for all monkeys together.

### One-trial learning: monkey alone

**Figure 2:**
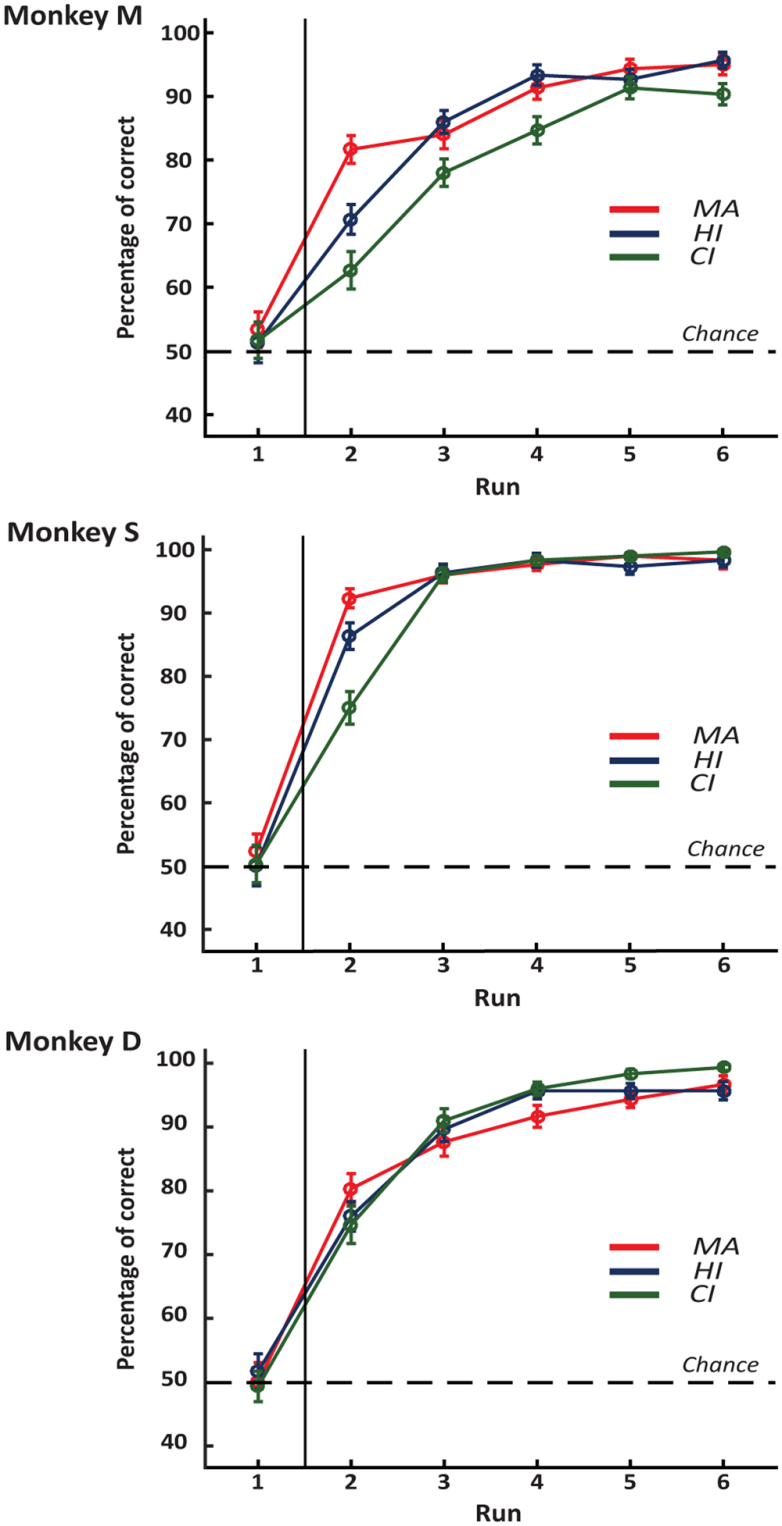
Learning curves for the three monkeys. The curves show the mean percentage of correct choices for the six runs in the 60 sessions for the three different conditions. Vertical bars represent the separation between runs always performed by the monkeys (on the right) and first runs performed by different agents depending on the experimental condition (on the left). Dashed lines represent chance level (50%). As expected, the first run performances do not differ from chance in any case. The mean percentage of correct choices in the second run is above chance in all conditions and for all three monkeys, showing that learning occurred after a single trial. Error bars represent standard error means (SEM).

We investigated the monkeys’ ability to learn the correct response of each problem maintaining or changing choice depending on the outcome of the trials of the first run. To evaluate learning, we calculated the percentage of correct responses in each run. As expected, in the MA version, the animals performed at chance level in the first run (exact binomial test: Monkey M, 53.3%, p = 0.273; Monkey S, 52.3%, p = 0.453; Monkey D, 50%, p = 1), choosing randomly one of the two objects (Fig. 2). Instead, all monkeys performed above chance in the second run (exact binomial test: Monkey M, 81.7%, p < 2.2e-16; Monkey S, 92.3%, p < 2.2e-16; Monkey D, 80.3%, p < 2.2e-16). This significant increase in the performance between the first and the second runs shows that monkeys learned from their choices during the first run improving their performance in the second run.

### One-trial learning: human and computer interactions

In the HI and CI versions, the human partner and the computer performed at chance level in the first run of the task (exact binomial test: HI and CI for Monkey M 51.3%, p = 0.686 and 51.7%, p = 0.603, respectively; HI and CI for Monkey S 50%, p = 1 and 50.3%, p = 0.954, respectively; HI and CI for Monkey D 51.7%, p = 0.603 and 49.3%, p = 0.862 respectively), likewise with the performance of the animals in the MA version. In the second run after the observation of the first run, the performance of all monkeys was significantly higher than chance level in both cases (exact binomial test: HI and CI for Monkey M, 70.7%, p = 5.788e-13 and 62.7%, p = 1.349e-05, respectively; HI and CI for Monkey S, 86.3%, p < 2.2e-16 and 75%, p < 2.2e-16, respectively; HI and CI for Monkey D, 76%, p < 2.2e-16 and 74.7%, p < 2.2e-16, respectively). These results show that the monkeys were able to learn after only one run also when they observed the task performed by another agent, either social or non-social.

However, the comparison of the monkeys’ performance in the three task versions showed a significant difference for Monkey M and Monkey S (one-way ANOVA, F (2,177) = 14.34, p = 1.7e-06 and F (2,177) = 17.37, p = 1.3e-07, respectively). A post hoc analysis revealed that the performance in the MA versions during the second run was significantly higher than in the HI and CI versions for Monkey M (MA vs HI, p = 0.0057 and MA vs CI, p = 2.9e-07, Tukey–Kramer multiple comparisons test), while for Monkey S the performance in CI was significantly lower than in MA and HI (MA vs CI, p = 2.04e-08 and HI vs CI, p = 4.34e-04, Tukey–Kramer multiple comparisons test). The performance during the second run showed no significant difference among the three versions of the task for Monkey D (one-way ANOVA, F (2,177) = 1.35, p = 0.26).

### Correct and errors

**Figure 3:**
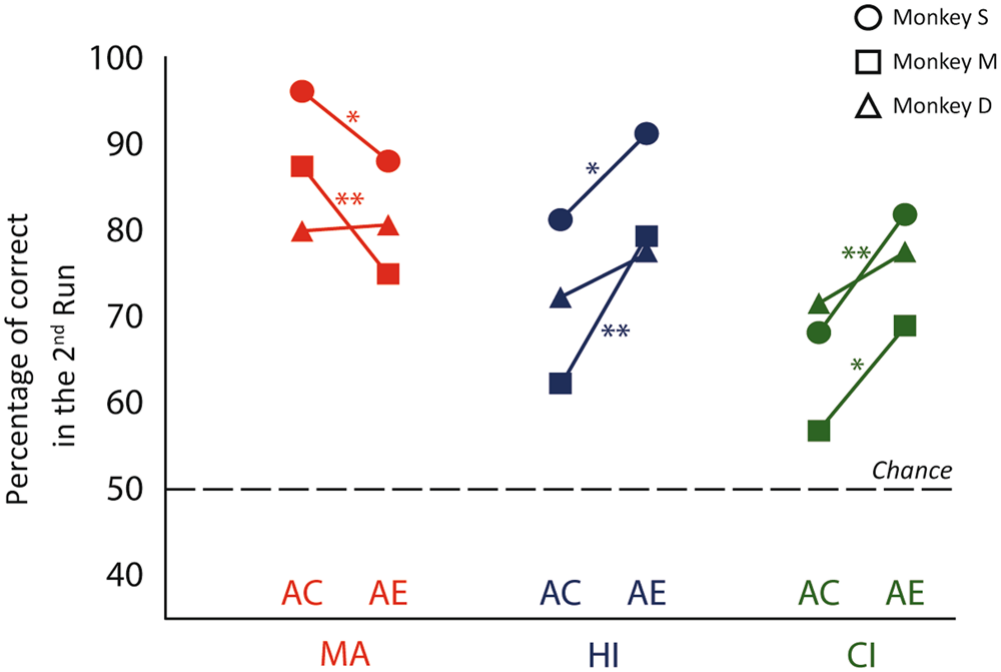
Performance in the second run after correct responses and errors made during the first run. The scatterplot shows the percentage of correct responses in the second run for the three monkeys in the three conditions, MA, HI, and CI. Trials were divided into two groups based on the performance in the first run, correct response or error. AC: after correct; AE: after error. Stars indicate significant differences (*p < 0.05 two-sample test for equality of proportions with continuity correction; **p < 0.01, two-sample test for equality of proportions with continuity correction).

We further investigated the consequences of successful or erroneous responses in the first run on the learning process by calculating the percentage of correct responses in the second run. The problems performed in the first run were divided into two groups: errors and correct choices. Supplementary Table S1 shows the monkeys’ performance in the second run separately for these two groups of problems. The scatterplot (Fig. 3) shows the percentage of correct trials in the second run in both cases, after a correct response or after an error in the first run, in the three versions of the task. In the MA version, two monkeys were significantly more accurate in the second run after a correct choice than after an error in the first run (two-sample test for equality of proportions: Monkey M, p = 0.008; Monkey S, p = 0.016), while for the third one that difference was not significant (two-sample test for equality of proportions: Monkey D, p = 1). In general, the learning in this condition was more effective after a first correct choice than after a first incorrect choice. Interestingly, in both interactive versions of the task, HI and CI, we observed the opposite pattern. Two monkeys were significantly more accurate in the second run after the observation of the human partner or the computer making an incorrect choice rather than a correct choice in the first run (two-sample test for equality of proportions: HI and CI for Monkey M, p = 0.001 and p = 0.039 respectively; HI and CI for Monkey S, p = 0.019 and p = 0.009 respectively). The third monkey showed the other monkeys’ tendency in the HI and CI conditions, although the difference was not significant (two-sample test for equality of proportions: HI and CI for Monkey D, p = 0.152 and p = 0.287 respectively). Altogether, the observational learning, social and non-social, allowed the monkey to reach better performance after the observation of an error rather than the observation of a correct choice. As a further control, we performed the same statistical analysis applying a Bayesian approach, using a Bayesian binomial test on the results in the first and in the second run, and implementing a Bayesian contingency table to test the difference between learning from correct and errors. The Bayesian method measures the Bayes Factor as a replacement of the p-values and the 95% credible intervals, enabling us to obtain a further confirmation of the strength of the results through a different approach (See Supplementary Methods, Supplementary Tables S3 and S4).

## Discussion

In this study we exploited a task design used to study one-trial learning in primate research. With different versions of the task, we assessed whether monkeys could benefit from the observation of an agent, social or non-social, in one-trial learning. The object-in-place paradigm^30,31^ has been used recently in primates to study rapid learning about the relative value of unfamiliar stimuli^32^. Alongside this, several studies demonstrated that rhesus macaques can monitor the action of another partner^5,21^ and learn from the observation of a conspecific^6,33^ or a human^9,20^. To compare social and non-social observational learning (i.e. human and computer interaction), we removed the ambiguity on the perception of the outcome that would differentiate the two conditions. Indeed, while the reward could be delivered to the human it cannot be delivered to the computer. Some studies, attempted to control the “social aspect” of the partner by placing an empty chair and dropping the reward on the floor^7,34^ or by showing the correct responses to the monkey via feedback without any delivery of reward^5^, but the success of these strategies on monkeys remains unclear. Here, we decided to deliver a reward to the monkey after the correct responses of both human and computer models in all cases, to facilitate the task monitoring and the learning of the correct object in all three conditions. In all 3 monkeys, we found evidence of one-trial learning from their own first choice, from the observation of the choice of a social human agent, and even from the observation of a non-social agent.

Previous studies reported similar learning abilities in the interaction with humans^9,20,26^. Monfardini *et al*.^9^ proposed that the perceived similarity between the observer and the model plays a crucial role in the enhancement of performance during an observational learning task similar to that observed in humans^35^. Critically, the observation of a human model simply showing the correct responses was not effective for generating learning by observation^6,9^. On the contrary, observing a “monkey like” acting human, who ignored the monkey and consumed the reward in the case of correct choices, improved the performance of the monkey after observation^9,20^. The vicarious aspect of the reward might seem critical to produce learning by observation. Indeed, in the studies that failed to report observational learning of non-human primates from human models^4,6^, the reward was not consumed by the model. However, this particular aspect of reward consumption falls short in our task, in which the monkeys consumed the reward after a correct response in all conditions. This difference between our study and past studies suggests that perceiving the others as similar might not be a prerequisite for observation learning. Alternately, the critical factor to promote the observational learning might be the evaluation of the outcome of an observed choice. In our case, monkeys directly experienced on themselves the outcome of another agent’s choices, i.e. the reward delivery or its absence in correct and incorrect trials, respectively. Consequently, learning by observation can occur through vicarious reward^9,26^ or, at least in the case of a one-trial learning, through non-vicarious reward: the key prerequisite is to provide a clear link between the observed choice and the outcome. In the previous works^9,26^, the association was made, as classically seen in the observational learning studies, between the behavior performed by the agent and the reward consumed by the agent. In our case, the association was made between the choice performed by the agent and the reward consumed by the observer, the monkey. The consumption of the reward, either by the monkey or the experimenter, could be the critical factor that promotes learning by observation through the generation of a clear choice-outcome association.

To maintain this association identical in both interaction conditions, we decided to apply the same paradigm in the computer condition. The computer’s choice – monkey’s reward consumption association was chosen to assess whether it is beyond the macaque ability to learn from observation from a non-physical agent, as previous studies seems to suggest, or if the past negative results of other studies were due to the inability to build such a clear association. Our main finding is that learning by observation occurred even with no real physical partner, in the computer interaction condition, also defined as a “ghost display” condition^36^. It has been demonstrated that human infants^37,38^ and children^28,36,39^ could learn in a ghost display condition. For example, 24-month-old children were able to replicate the actions of an “invisible agent” in a push or pull bidirectional task, after the simple observation of the door of the box moving by itself^29^. However, great apes in the same paradigm were able to open the door of the box after the observation of the “ghost display” condition but were unable to match their actions with the previously observed one of the “invisible agent”. Hopper *et al*.^27^ evaluated chimpanzees in the ghost condition with an apparatus that either could be lifted or poked to receive a food reward. Learning occurred only when the animals observed a conspecific already trained to use the apparatus, before their own session. On the other hand, performance remained at chance when they observed the automatic movement of the device in the ghost condition. The same authors^28^ replicated an experimental paradigm previously used with birds^15^ in which the door of a box containing the reward was moved in a ghost condition. In this situation, chimpanzees were able to extract the relevant information and match their movement with that of the “invisible agents”, but only in the first responses. The authors explain this partial positive result compared to previous studies as being a direct consequence of the easy task requirements. More complex tasks, involving more sophisticated motor acts and/or the utilization of tools, are more demanding and seem more challenging to be learned by observation in the ghost display condition. If the complexity of the task requirements plays such an important role in the inability of monkeys to learn, it should not be surprising that learning abstract cognitive rules is beyond the bounds of possibility in ghost display conditions. Indeed, to our knowledge, the only study which tried to establish such cognitive learning in a ghost display condition in rhesus macaques^5^ failed to find evidence of it. Subiaul *et al*.^5^ trained two macaques to learn to respond in a prescribed order to a set of pictures displayed on a touchscreen. In the social learning condition, the monkeys learned a new sequence by observation of an “expert” monkey. On the other hand, in a “ghost display” condition (i.e. the observation of a monitor in which the task was carried on automatically, with all the auditory and visual feedback) there was no learning without the “expert” monkey working. Therefore, monkeys were able to learn by observation of a conspecific but not by observation of the correct sequence displayed automatically. In our task, the monkeys were trained to let the computer work automatically for the first run and observed the visual feedback and consumed the reward in the correct cases (50% of the trials approximately). In the second run, although generally to a lesser extent than in the MA and the HI conditions, the performance in the CI condition increased significantly over chance. To account for the differences between the results of the two experiments, we must consider three main factors. First, in the study of Subiaul and colleagues^5^, monkeys worked on two different touchscreens in two different chambers separated by a transparent glass, side by side. During the “ghost display” condition, the touchscreen of the observer monkey was inactivated, and the monkey could monitor and learn from the observation of the touchscreen in the other chamber, where the second monkey was previously sitting. The lack of learning could be accounted for by the difficulty generated by that particular setting to attract attention to the events occurring in the next chamber in the absence of a real physical agent inside. We overcame that limitation, in our experiment running both human and computer interactive conditions on the same touchscreen where the monkey worked alone. The touchscreen was placed in front of the monkey in every condition and its head was fixed, therefore its attention on the task was maximized in all three conditions. Second, a fundamental difference between our task and the work of Subiaul *et al*.^5^ is the reward delivery. With our task design the monkeys received the reward in the two observational learning conditions after a correct response. The positive feedback was combined with the reward delivery in the case of correct responses while the negative feedback was not followed by reward. Instead, in the study of Subiaul *et al*.^5^, monkeys could rely only on the auditory and visual feedback associated with the correct responses without receiving the reward. The mere presence of the feedback in the previous study^5^ could not have been sufficient to enhance the performance in the “ghost display” condition. Third, the object-in-place task design is peculiar. The redundancy of information available in a typical scene, such as the background colour, the shapes forming the scene, the position of the objects or the shape of the objects themselves play a crucial role and enhance the learning, making the information much easier to remember. This paradigm is indeed used to generate one-trial learning processes. The classical Pavlovian conditioning and the instrumental conditioning are usually studied with behavioral tasks which require more trials to reach a learning criterion. We considered the difference between our paradigm and a Pavlovian-instrumental transfer in the Supplementary Discussion. The particularity of the OIP task lies in the fact that the learning process is facilitated by the presence of the background scene and the first observed or performed choice can be easily monitored to enhance the performance already on the second run of the task. In this task the background scene may be important for one-trial learning because it might have the function of a retrieval cue for each previous encountered scene such that its absence in conditional discrimination tasks prevent it^30^. This particularity could explain why in our case, the monkeys were able to learn in the ghost display condition. During the human and computer interaction conditions, we did not control the position of the hand of the monkey when observing. However, we did not observe systematic arm movement during these trials (e. g. movement directed to the touchscreen without touching it) that could provide an alternative interpretation of our results.

Our second finding is that different mechanisms underlie individual learning and observational learning: monkeys learned more from their own correct choices and more from the observation of other’s mistakes. Other studies have already reported similar results in humans^8^, monkeys^8,9,33^ and birds^13^ demonstrating that learning from others’ errors is easier than from their successes. Some studies highlighted and explained that this difference is likely due to the choice-induced preference^8,9^. In psychology, cognitive dissonance theory assumes that human and non-human primates look for internal consistency^40–42^. Consequently, individuals value more the alternative they have precedingly chosen and encounter some difficulties in correcting personal mistakes. This is in line with errorless learning theory^43^, claiming that errors are not necessary and discrimination learning does not require responses to the incorrect object. On the other hand, it has been shown that observing the lack of success of others is more informative and it is easier to learn after error^8,13,44^. A hypothesis to explain this facilitation is based on gloating that refers to our own satisfaction of observing others’ troubles. Gloating can explain the influence of such observation on later choices and the capacity to learn from other mistakes in humans^45^. However, the existence of such a mechanism has never been proved in non-human primates and that explanation could be ruled out by our paradigm because the reward was also obtained after others’ correct actions. Moreover, in the present study we showed a similar learning after errors of social and non-social agents suggesting that the underlying mechanisms are comparable. Indeed, in the computer interaction, we observed the same effect as in the human interaction condition: observing computer mistakes appeared to be more informative for the monkeys than observing computer correct choices.

The study of the neural bases of social interactions and more precisely the monitoring of other choices and social learning is a current topic of interest. Brain responses during individual and observational learning have been studied and revealed a common network for these two types of learning^46^. However, recent studies have emphasized the difference in the neural substrates between self and others at the single cell level, not only at the level of action and choices^18,19,23–25^ but also in terms of errors responses both in monkeys^47,48^ and in humans^49^ indicating a different neural processing of errors. The present study sheds light on the existence of a one-trial observational learning from a non-social agent, in the so-called “ghost display” condition in macaque monkeys and creates a window of opportunity to better understand the neurophysiological substrates of observational learning. Although further work is needed to better understand the specificity of learning from social versus non-social agents, our work suggests that there should be a general reassessment of the idea that macaques cannot learn in the ghost condition.

## Materials and Methods

### Animals

Three male rhesus monkeys participated in this study: Monkey M (5 years and 10 months old, 7.5 kg), Monkey S (6 years and 2 months old, 8.00 kg) and Monkey D (6 years and 8 months old, 7.5 kg). Animal care, housing, and experimental procedures conformed to the European (Directive 210/63/EU) and Italian (DD.LL. 116/92 and 26/14) laws on the use of non-human primates in scientific research. The research protocol was approved by the Italian Health Ministry (Central Direction for the Veterinary Service).

### Surgical techniques

In the three monkeys, the experiments were carried out while the monkey’s head was fixed. For this purpose, we implanted a head-holder. Each animal was preanesthetized with ketamine (10 mg/kg, i.m.) and anesthetized with isoflurane (Abbott Laboratories) through a constant flux of isoflurane/air mixture (1–3%, to effect). Antibiotics and analgesics were administered postoperatively.

### Apparatus

Monkeys sat in a primate chair with the head fixed in front of a monitor touch screen (3M™ MicroTouch™ M1700SS 17” LCD touch monitor, 1280 × 1024 resolution). A non-commercial software package, CORTEX (NIMH, Bethesda, USA) was used to display and control stimuli on the touch screen, to deliver reward, and to record the monkey’s behavioral responses. After each correct trial, the animals received apple sauce as a reward.

### Task: scenes, objects and problems

In the object-in-place (OIP) task we wanted to assess the first stage of a learning process, that is one-trial learning^30–32^. It has been also named object-in-place scene-learning task^50^. During the OIP discrimination learning, two objects, one rewarded and the other unrewarded, were always displayed at the same places within a unique background, the scene, and formed the problem that the monkeys had to solve.

A *scene* was composed by a random color background, and three geometrical figures, randomly selected from a list of 200 possible items. An *object* consisted of two superimposed colored ASCII characters pseudorandomly generated (Fig. 1A left). The randomization of the colored background, the geometrical figures and the objects, allowed the display of unique patterns on the screen and the creation of unique problems to solve. Each of the 60 sessions per condition performed by the monkeys included five different unique problems, which were not repeated in the successive sessions. In this way, during the first run of each session, were presented five different problems never encountered before.

### Task: trial structure

A trial began with a central target (CT) presented on the screen, represented by a white circle (Fig. 1B). The monkey had to touch the CT and hold the contact for 0.4 or 0.6 s to let the scene and the objects appear. After a delay period of 0.8 or 1.2 s, the CT disappeared, indicating the go signal for the monkey to touch one of the two objects displayed in the scene. Then, after the monkey maintained the touch for a pre-feedback period (0.4 or 0.6 s) a visual feedback surrounding the chosen object was presented. A white square or a red hexagon indicated a correct response, and a white circle or a blue hexagon indicated an error (Fig. 1A right). This visual feedback remained on the screen for a holding period (0.4 or 0.6 s) during which the monkey was required to hold the hand on the target. After a correct response, a reward was delivered and then the screen turned black, while after an error no reward was provided, and the scene and the objects turned off. If the monkey aborted the trial before the feedback appearance, the same problem was presented again. If a trial was executed until the end, the next problem was presented in the successive trial even after an error, without a correction trial.

### Task: runs

Five different problems were presented consecutively to the animal, repeated for six runs, for a total of 30 trials (Fig. 1C). During the first run, monkeys had no clue about which one of the two objects was the rewarded one, they had to guess and randomly select one of them. After five problems, the first run ended, and the second run started with the presentation of same five problems in the same order. The session ended after the monkey completed six runs.

### Task conditions

Monkeys performed three different versions of the OIP task: a *monkey alone* (MA) version, and two “interactive” versions: a *human interaction* (HI) version and a *computer interaction* (CI) version. With these task versions we could study individual learning, social observational learning, and non-social observational learning (from an external and non-social agent). In the MA version, the monkeys performed all six runs by themselves. In the HI version, the experimenter sat close to the monkey and faced the monitor. The time course of the task was identical except that the human performed the first run and the monkey observed without intervening. After the end of the first run, the monkey could complete the remaining runs (from the second to the sixth run). The human in the first run chose one of the two objects randomly therefore reaching chance level, resembling the monkeys’ behavior in the first run of the MA condition (see Supplementary Fig. S1). The monkey received the reward only after a correct human choice and not after an error.

Finally, we carried out the CI version to test the non-social observational learning (by observation but in the absence of a real partner). Like in the HI version, the monkeys had to observe the first run and complete the remaining ones. Instead of a white CT, they recognized the appearance of a red CT in the first run as a stop signal. If the monkeys did not touch the screen, the computer trial continued, and the problem appeared on the monitor. Thus, the computer pseudorandomly performed the choice of one of two objects, selecting either the correct one or the incorrect one in equal proportions, similar to the HI and MA conditions (see Supplementary Fig. 1). All the periods of the trial were identical to those of the other two versions, except for a randomly defined dummy time simulating the reaction times and the movement times of monkeys and humans (randomly selected between 650 and 950 ms). Then, the position and the type of visual feedback around one of the two objects informed the monkey about the computer choice. A reward was delivered to the animals after a correct computer choice and not after an error. Trials were aborted and restarted whenever the monkey touched the screen during any period of the computer trial. At the end of the first run, the trials began with the white CT and the monkeys performed the remaining five runs. The appearance of the white CT in the second run was interpreted by the monkeys as a stimulus to touch and they were able to shift from the first run made by the computer to the second run performed by themselves without any subsequent training.

### Training

Monkeys were trained in the preliminary versions of the task on a daily basis before starting to collect the data for the experiment. Several features of the task were first tested, the number of successive problems, the complexity of the scenes, the temporal sequence of the events within a trial, and the use of feedbacks. We started to collect the monkeys’ behavior after they acquired sufficient familiarity with the task schedule in all three versions. Monkey M and Monkey S were trained for approximately one and a half months before we collect the data presented here. Monkey D went through an additional period of approximately three months of training, in order to start the recording sessions after complete recovery from the surgical implantation of the head-holder. In most of the cases there was no improvement in one-trial learning over the sixty recorded sessions (see Supplementary Fig. S2 and Supplementary Table S2). For the CI condition, monkeys were trained to recognize the appearance of a red CT instead of a white one as a stop signal for the execution of the trial. In the early phases of the training, the red CT was presented alone on the screen and monkeys were trained to not touch it in order to received reward after few seconds. A touch of the red CT automatically led to an abort of the trial without the reward delivery and to the representation of another trial with a red CT. We gradually extended the time from the appearance of the red CT and the delivery of the reward until the monkeys were used to wait without trying to touch. After this phase, we presented the complete trial performed by the computer.

Monkey S and Monkey M performed the sixty sessions of the MA and the sixty sessions of the HI conditions in the same days, alternating between blocks of four sessions per condition. Afterwards, were carried out the sixty sessions of the CI condition. Monkey D performed first the sixty sessions of the MA condition. Afterwards, the sixty sessions of each of the two interactive conditions, HI and CI, were performed by monkey D during the same days, alternating between blocks of four sessions per condition.

## Electronic supplementary material

Supplementary information

## Data Availability

The dataset generated and/or analysed during the current study is available from the corresponding author on reasonable request.
