# Supplementary material for: Macaque monkeys learn by observation in the ghost display condition in the object-in-place task with differential reward to the observer
Source: Sci Rep. 2019 Jan 23;9:401. doi: 10.1038/s41598-018-36803-4 (PMC6344553; doi:10.1038/s41598-018-36803-4)
Supplement: Supplementary file 1 — Supplementary information [file 41598_2018_36803_MOESM1_ESM.pdf]

Supplementary Information

Title: Macaque monkeys learn by observation in the ghost display condition in the object-in-place task with differential reward to the observer

Lorenzo Ferrucci[1,2], Simon Nougaret[1], Aldo Genovesio[1*]

[1] Department of Physiology and Pharmacology, Sapienza University of Rome, Rome, Italy

[2]PhD program in Behavioral Neuroscience, Sapienza University of Rome, Rome, Italy

Corresponding author:

Prof. Aldo Genovesio

Department of Physiology and Pharmacology, Sapienza University of Rome, Rome, Italy

Tel. +39 06 49910753

e-mail: aldo.genovesio@uniroma1.it

		2ND RUN	
	**1ST RUN**	**Corrects**	**Errors**
**Monkey M MA**	Corrects (160/300)	140/160	20/160
	Errors (140/300)	105/140	35/140
**Monkey M HI**	Corrects (154/300)	96/154	58/154
	Errors (146/300)	116/146	30/146
**Monkey M CI**	Corrects (155/300)	88/155	67/155
	Errors (145/300)	100/145	45/145

		2ND RUN	
	**1ST RUN**	**Corrects**	**Errors**
**Monkey S MA**	Corrects (157/300)	151/157	6/157
	Errors (143/300)	126/143	17/143
**Monkey S HI**	Corrects (150/300)	122/150	28/150
	Errors (150/300)	137/150	13/150
**Monkey S CI**	Corrects (151/300)	103/151	48/151
	Errors (149/300)	122/149	27/149

		2ND RUN	
	**1ST RUN**	**Corrects**	**Errors**
**Monkey D MA**	Corrects (150/300)	120/150	30/150
	Errors (150/300)	121/150	29/150
**Monkey D HI**	Corrects (155/300)	112/155	43/155
	Errors (145/300)	116/145	29/145
**Monkey D CI**	Corrects (148/300)	106/148	42/148
	Errors (152/300)	118/152	34/152

Supplementary Table S1. Performance in the first and second run for each monkey (related to Fig. 2 and 3). The 1st Run column shows the number of correct and error trials in each condition, on the total of 300 trials (5 trials x 60 sessions). The 2nd Run columns shows the proportions of correct and error trials divided for the two groups, Corrects in the 1st run and Errors in the 1st Run. The numbers in the 2nd Run – Corrects columns are reported as a percentage of correct trials in the scatterplot of Fig. 3. Abbreviations: MA, Monkey Alone; HI, Human Interaction; CI, Computer Interaction.

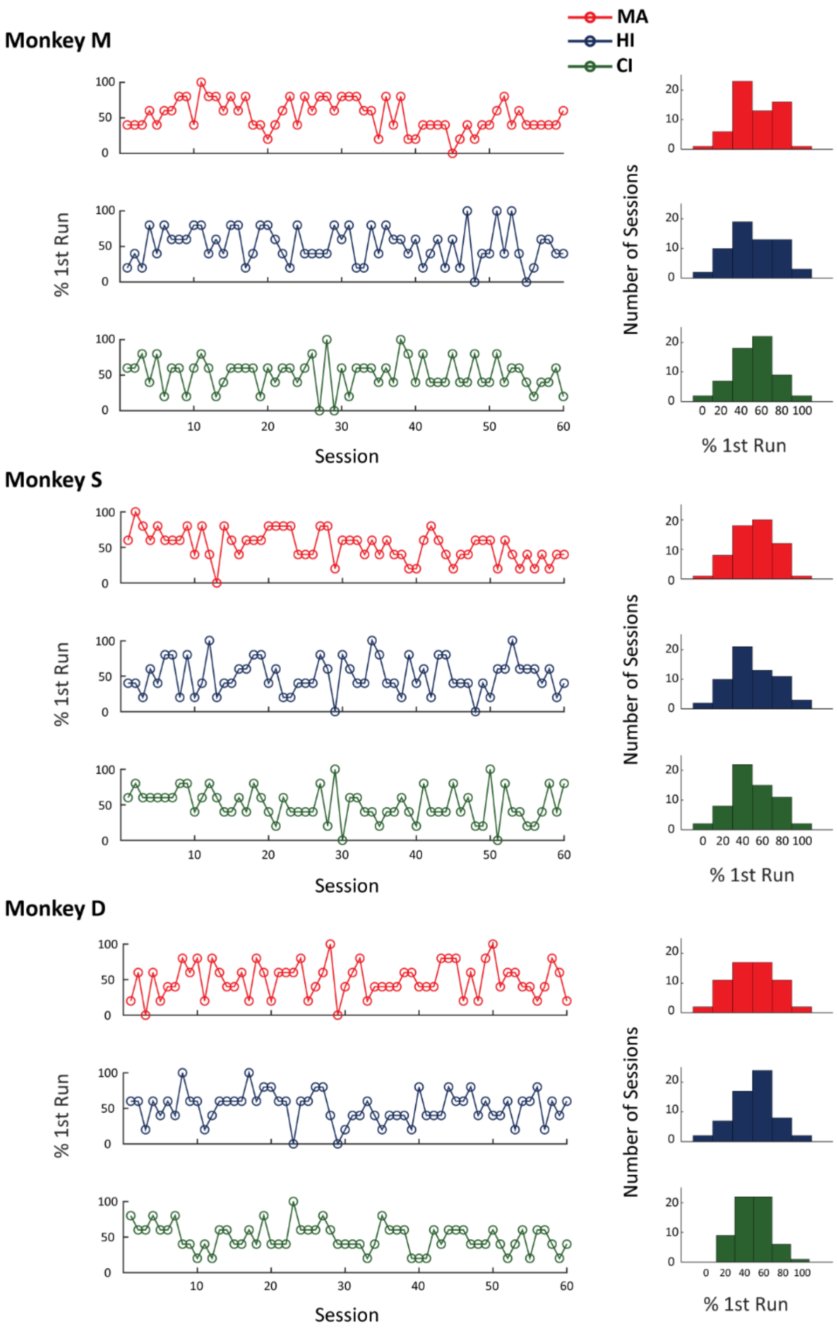

Supplementary Figure S1. Time course of the tasks performance in the first run in the three experimental conditions over the sixty sessions (Related to Fig. 2). In the MA condition, the first run was performed by the monkey, in the HI condition, the first run was performed by the human agent and in the CI condition, the first run was performed automatically by the computer. Bar graphs represent the distribution of the percentage of correct responses in the first run in the three conditions. There was no significant difference between the distribution of the percentage of correct trials between the MA and the HI conditions and between the MA the CI conditions for all three monkeys (Wilcoxon Rank-Sum Test, $p > 0.05$). Abbreviations: MA, Monkey Alone; HI, Human Interaction; CI, Computer Interaction.

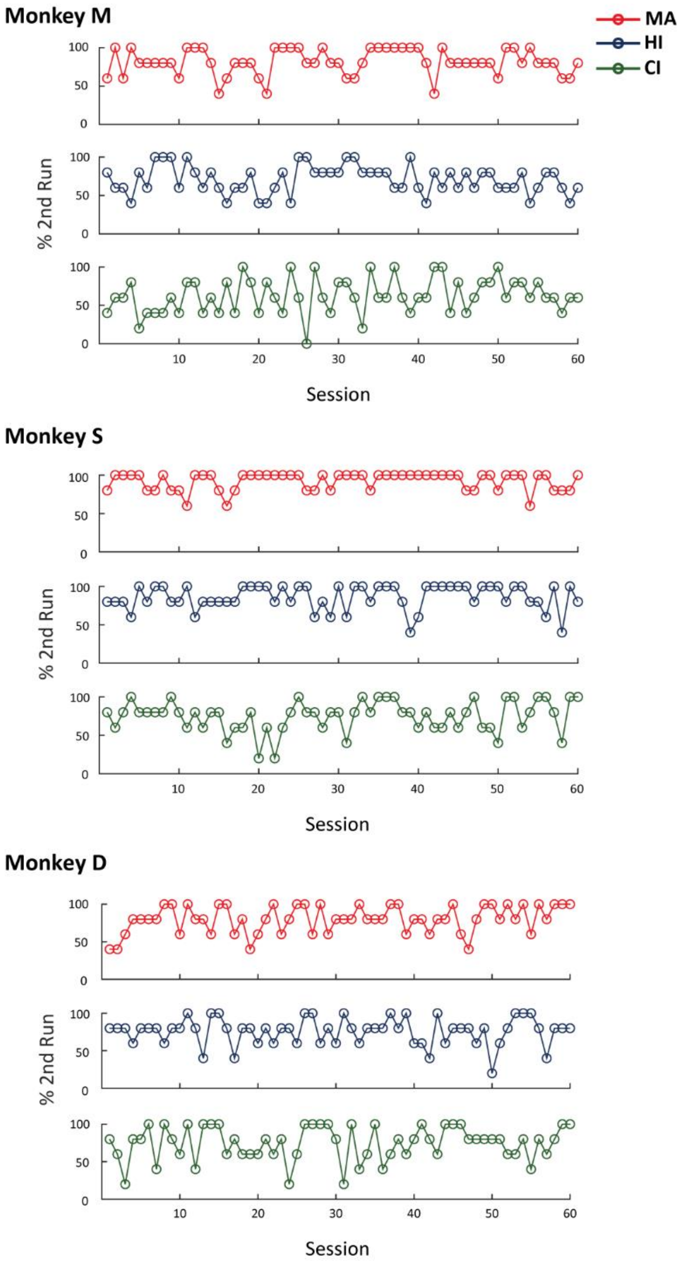

Supplementary Figure S2. Time course of the monkey's performance in the second run in the three experimental conditions over the sixty sessions (Related to Fig. 2). In 8 out of 9 cases, there was no significant correlation between the monkey's performance and the number of session (see Supplementary Table 2). Abbreviations: MA, Monkey Alone; HI, Human Interaction; CI, Computer Interaction.

	MA	HI	CI
Monkey M	P=0.7650	P=0.3384	P=0.0873
	R=0.0394	R=-0.1257	R=0.2227
Monkey S	P=0.6001	P=0.7002	P=0.3472
	R=0.0689	R=0.0507	R=0.1235
Monkey D	P=0.0340	P=0.7994	P=0.4345
	R=0.2743	R=-0.0335	R=0.1028

Supplementary Table S2. Pearson's Linear Correlation coefficients and corresponding p-values between the performance in the second run for each condition and the session's number. Only Monkey D showed a significant correlation in the MA condition. Abbreviations: MA, Monkey Alone; HI, Human Interaction; CI, Computer Interaction.

Supplementary Method

We computed a Bayesian binomial test to compare the proportion of correct trials during the first and the second run for each monkey in each experimental condition, with H_0=0.5 and $H_1 \neq 0.5$ (Supplementary Table S3). The Bayesian approach allows to compare the likelihood of the data under both the null and the alternative hypothesis, bypassing some of the major issues related to the interpretation of p values which occur using frequentist approaches (Wagenmakers 2007). The observed values are expressed in terms of likelihood function of the data and are weighted with a prior distribution, that is the probability distribution of the data before the observation happens. We made no assumptions on the prior distribution ($\alpha=\beta=1$). Bayes factor is reported as the ratio between the likelihood of the data under H_1 and the likelihood of the data under H_0 (BF_{10}). BF_{10} >1 indicates that data of the posterior distribution are more likely under H_1. The 95% credible interval are also reported. They represent the 95% probability that the performance falls between these specific upper and lower intervals. We tested the difference in the proportion of correct trials in the second run using a Bayesian Contingency Tables Test (Supplementary Table S4). We divided the trials performed in the second run in each condition by each monkey in two groups, based on the correct (Group 1) or incorrect (Group 2) responses given for those trials in the first run. Since the marginal distribution in the contingency tables was fixed (300 trials) in each one of the tested condition we applied a joint multinomial sampling scheme and we computed the BF_{10} joint multinomial given the null hypothesis Group 1 = Group 2 and the alternative hypothesis Group 1 ≠ Group 2. Furthermore, we computed the Log Odds Ratio (Log OR) and its 95% credible intervals to address the direction and the strength of the effect. Log OR > 0 indicates that a correct answer in the second run is more likely in Group 1, and viceversa. These analyses were performed using JASP Team (2018). JASP (Version 0.9) .

Bibliography

Wagenmakers, E., Lee, M., Lodewyckx, T. & Iverson, G. *Bayesian Versus Frequentist Inference*. (2008). doi:10.1007/978-0-387-09612-4

Condition	Monkey	Run	BF_{10}	95% C.I.
MA	Monkey M	1st	0.14	0.48-0.59
		2nd	9.675e+26	0.77-0.86
MA	Monkey S	1st	0.1	0.47-0.58
		2nd	4.415e+53	0.89-0.95
MA	Monkey D	1st	0.07	0.44-0.56
		2nd	3.006e+24	0.75-0.84
HI	Monkey M	1st	0.08	0.46-0.57
		2nd	1.941e+10	0.65-0.75
HI	Monkey S	1st	0.07	0.44-0.56
		2nd	1.09e+37	0.82-0.90
HI	Monkey D	1st	0.08	0.46-0.57
		2nd	1.995e+17	0.71-0.80
CI	Monkey M	1st	0.08	0.46-0.47
		2nd	1176.96	0.57-0.68
CI	Monkey S	1st	0.07	0.45-0.56
		2nd	6.909e+15	0.70-0.80
CI	Monkey D	1st	0.07	0.44-0.56
		2nd	2.334e+15	0.69-0.79

Supplementary Table S3. Bayesian binomial test results

Condition	Monkey	BF_{10} Joint Multinomial	Log Odds Ratio	Log OR 95% C.I.
MA	Monkey M	7.960	0.834	[0.262,1.434]
MA	Monkey S	3.507	1.150	[0.264,2.139]
MA	Monkey D	0.173	-0.033	[-0.589,0.536]
HI	Monkey M	39.76	-0.840	[-1.359, -0.333]
HI	Monkey S	3.529	-0.858	[-1.566,-0.189]
HI	Monkey D	0.622	-0.422	[-0.967,0.097]
CI	Monkey M	2.224	-0.520	[-0.995,-0.062]
CI	Monkey S	7.777	-0.742	[-1.281,-0.200]
CI	Monkey D	0.380	-0.313	[-0.839,0.200]

Supplementary Table S4. Bayesian Contingency Table Test results

Supplementary Discussion

The aim of our work was to test whether macaques could learn from what we called a “non-social” agent. We contrast our result with the negative finding of Subiaul and colleagues in 2004 who failed to find any evidence of learning in a ghost display condition in macaque’s monkeys. We hypothesized that an important difference between the social observational learning condition and the ghost display condition in their study might be that the monkeys did not associate the perceived action with the reward in the ghost display condition. Indeed, in their social observational learning condition, the other monkey obtained the reward while in their ghost display condition there was no reward at all. For this purpose, we decided to have two comparable conditions delivering the reward to the monkey in both cases. However, we are aware of the risk that our observational learning condition became a Pavlovian (1st trial)-instrumental (2nd trial) transfer (PIT). We can think to three main arguments against that interpretation.

First, the Pavlovian part of a PIT would require a classical conditioning learning. Some previous work described the possibility to create a fast Pavlovian association in a few trials (Rescorla 1988) but most of the paradigms used in the literature included several sessions of classical conditioning before testing the transfer to instrumental conditioning. Moreover, it is usually an increase in the performance which is measured and not the capacity to make a choice between two objects.

The second argument refers to the task requirement of making a choice. In our versions of the object-in-place task, correct and errors were made in the same proportion by the human partner or by the computer in the first run. Consequently, in 50% of the cases, the monkey had to apply a loose-shift strategy, shifting from the incorrect object chosen by the other agent in the first run to the alternative object in the second run. Because in this 50% of cases the animal was not exposed to the positive feedback and to the reward and it can be ruled out a simple Pavlovian conditioning learning effect. The negative feedback was also presented over the chosen object, not over the correct one. In case of incorrect choices, the correct object (the unchosen one) was not associated with any feedback, precluding any Pavlovian conditioning learning effect. In addition, it is precisely the error made by the human or the computer that promoted the higher learning effect in the observation conditions and the monkey received the reward after observing a correct choice but not after an observed incorrect choice. It means that our monkeys were able to link the action made by the human or the computer with a positive (reward) or a negative (absence of reward) outcome by observation.

Finally, the third argument refers to the peculiarity of one-trial learning processes. It has been shown that this kind of learning relies on the activity of high level prefrontal areas such as the frontal pole (FP). Indeed, in monkeys, lesions of the FP cause a specific impairment on the learning rate in the OIP task (Boschin 2015) with the loss of the one-trial learning effect. Moreover, this fast learning seems dependent of prefrontal processes that unites all information about the different features of a scene. Such processes require high level integration and differ from Pavlovian conditioning.

Bibliography

Rescorla, R. A. Pavlovian conditioning it’s not what you think it is. *Am. Psychol.* 43 (3):151-60 (1988)

Boschin, E. A., Piekema, C. & Buckley, M. J. Essential functions of primate frontopolar cortex in cognition. *Proc. Natl. Acad. Sci.* 112, E1020–E1027 (2015).
